# Interaction analyses based on growth parameters of GWAS between *Escherichia coli* and *Staphylococcus aureus*

**DOI:** 10.1186/s13568-021-01192-x

**Published:** 2021-03-01

**Authors:** Yajing Liang, Beibei Li, Qi Zhang, Shilong Zhang, Xiaoqing He, Libo Jiang, Yi Jin

**Affiliations:** 1 Beijing Advanced Innovation Center for Tree Breeding by Molecular Design, Beijing Forestry University, Beijing, 100083 China; 2 Center for Computational Biology, College of Biological Sciences and Technology, Beijing Forestry University, Beijing, 100083 China; 3 College of Biological Sciences and Technology, Beijing Forestry University, Beijing, 100083 China

**Keywords:** Interaction mechanism, Growth parameters, Growth trajectories, Significant SNPs

## Abstract

To accurately explore the interaction mechanism between *Escherichia coli* and *Staphylococcus aureus*, we designed an ecological experiment to monoculture and co-culture *E*. *coli* and *S*. *aureus*. We co-cultured 45 strains of *E*. *coli* and *S*. *aureus*, as well as each species individually to measure growth over 36 h. We implemented a genome wide association study (GWAS) based on growth parameters (*λ, R, A* and *s*) to identify significant single nucleotide polymorphisms (SNPs) of the bacteria. Three commonly used growth regression equations, Logistic, Gompertz, and Richards, were used to fit the bacteria growth data of each strain. Then each equation’s Akaike’s information criterion (AIC) value was calculated as a commonly used information criterion. We used the optimal growth equation to estimate the four parameters above for strains in co-culture. By plotting the estimates for each parameter across two strains, we can visualize how growth parameters respond ecologically to environment stimuli. We verified that different genotypes of bacteria had different growth trajectories, although they were the same species. We reported 85 and 52 significant SNPs that were associated with interaction in *E. coli* and *S. aureus*, respectively. Many significant genes might play key roles in interaction, such as *yjjW*, *dnaK, aceE*, *tatD*, *ftsA*, *rclR*, *ftsK*, *fepA* in *E. coli*, and *scdA, trpD, sdrD*, SAOUHSC_01219 in *S. aureus*. Our study illustrated that there were multiple genes working together to affect bacterial interaction, and laid a solid foundation for the later study of more complex inter-bacterial interaction mechanisms.

## Introduction

There are extremely complex interactions between organisms and their environment, which play a crucial role in nature, particularly in microbial environments (Grativol et al. 2017; He et al. 2017; Madsen et al. 2018; Pires et al. 2015; van Overbeek and Saikkonen 2016). A large number of strains exist in co-cultured complexes of bacteria (Cairns et al. 2018), and they form diverse and dynamic communities with complex interaction mechanisms including mutualism, antagonism, parasitism, commensalism, and amensalism (Widder et al. 2016; Xiao et al. 2017). Microbial interactions have rarely been experimentally validated due to the challenges of duplicating ecologically relevant conditions in a laboratory environment and the limited ability to culture all members of a complex multispecies microbiome (Kastman et al. 2016). Therefore, we constructed a simplified experimental system to simulate culture conditions.

A genome-wide association study (GWAS) with longitudinal phenotypes provides a novel platform to identify genetic variant associations and how they change over time (Sikorska et al. 2018). The emergence of GWAS has accelerated the development of bacterial gene function research, which enables the screening of interaction-associated loci (Lees et al. 2016; Ning et al. 2017; Sheppard et al. 2013). Therefore, the application of GWAS to bacteria opens new horizons for exploring the interaction mechanisms of bacteria. Many bacterial phenotypes can be linked to the presence or absence of significant genes, which partly explains different responses to similar environmental conditions. There is great potential to investigate environmentally or industrially relevant phenotypes as well (Chen and Shapiro 2015). Recently bacterial GWAS have overcame some limitations of the traditional genetic association studies, such as sample sizes and population structure, which is able to associate genetic variation in genomes with phenotypes to analyze genetic mechanisms (Lees et al. 2016). Studies on single-nucleotide polymorphism (SNPs) are a starting point for identifying genes that may be responsible for specific phenotypes (Hall 2014). In our lab, we have applied GWAS analyses to find many significant genes in *E. coli* and *S. aureus* and have explored the phenotypic plasticity of *S. aureus* (He et al. 2017; Jiang et al. 2018; Rong et al. 2019). Our analyses provide a strong basis for interaction experiments of bacteria in future studies.

Currently most GWAS methods are based on simple genotype phenotype analyses, and dynamic growth is treated statically. Gene–gene and gene-environment interactions are time dependent and temporal dynamical interactions require efficient modeling (Fan et al. 2012). Complex phenotypes arise as a function of time, so we cannot capitalize on full information of phenotypic expression. A previous study integrated GWAS and functional aspects of dynamic traits, proposing a novel statistical approach called functional GWAS (*f*GWAS) (Das et al. 2011). It is able to address the limitations of traditional GWAS methods, and uses growth trajectories as a phenotype to conduct GWAS, which remarkably increases the power for gene detection. In this way, *f*GWAS can capture genotypic differences at the level of phenotypic curves according to growth parameters (Li et al. 2015; Wei et al. 2018). On this basis, a previous study fit growth equations to leaf area and leaf area mass growth data of an individual recombinant inbred line (RIL), and developed a bivariate model for mapping growth parameters of the two traits (Wei et al. 2018).

In this study, we extended the application of GWAS based on growth parameters to the study of bacterial interactions, applying functional mapping and statistical analyses to express bacterial growth phases. Then we simulated the living environment of *E*. *coli* and *S*. *aureus* to study their dynamic interactions and to target phenotypic traits. The aim of our study is to explore the genetic mechanism of bacterial interaction, therefore, lay a scientific theoretical foundation for microbial community analyses.

## Materials and methods

### Experimental design

We applied statistical analyses to study the growth interactions of *E. coli* and *S. aureus* in a common environment and explore important gene mechanisms. Based on co-cultures, we implemented a bivariate statistical procedure to map and identify SNPs. Using these SNPs, we predicted bacterial growth by estimating cultivar-specific growth parameters and incorporating these parameters into a mapping framework.

All the strains of *E. coli* and *S. aureus* are from National Infrastructure of Microbial Resources, China, and these are detailed in previous work (Jiang et al. 2018) (Additional file 1: Table S1). We separately monocultured and co-cultured the strains under different conditions, although temperature, culture time, and environment were kept constant.

We investigated relationships by measuring the abundance of each strain repeatedly at multiple times. We randomly paired 45 strains of *E. coli* and 45 strains of *S. aureus*, and cultured each pair in a 50 mL Erlenmeyer flask with three parallel treatments, following a previous study (Jiang et al. 2018). Then we obtained the growth data of the strains in monoculture and co-culture as phenotype data (Additional file 2: Table S2).

### Whole-genome sequencing

Whole-genome sequencing was performed on an Illumina HiSeq2000/2500 platform at Novogene (Novogene, Beijing, China) using *E. coli* str. K-12 substr. MG1655 and *S. aureus* subsp. *aureus* NCTC 8325 as the reference strains, respectively. More details can be found in a previous study (He et al. 2017).

### Data fitting

Bacterial growth follows a standard s-shaped curve. A typical growth curve spans three continuously connected phases: the lag phase (where the growth rate emerges from a value of zero), the exponential phase (during which the rate of growth accelerates to a maximal value and then decelerates to a minimal value, cells are typically in their healthiest state and thus are most desirable for enzymes or other cell components), and the stationary phase (growth rate continues to decelerate to zero and bacteria reached a stable state). Many mathematical equations have been derived to reflect the features of a growth curve, including Gompertz, logistic, and Richards equations. Let *g*(*t*) denote the growth of a trait at time *t*. These equations are expressed as

$$ g\left( t \right) = \left\{ {\begin{array}{*{20}l}    {A \cdot \exp \left\{ { - \exp \left[ {\frac{{R \cdot e}}{A}\left( {\lambda  - t} \right) + 1} \right]} \right\}} & {{\text{Gompertz}}}  \\    {\frac{A}{{1 + \exp \left[ {\frac{{4R}}{A}\left( {\lambda  - t} \right) + 2} \right]}}} & {{\text{Logistic}}}  \\    {A\left\{ {1 + s \cdot \exp (1 + s) \cdot \exp \left[ {\frac{R}{A}(1 + s)^{{\left( {1 + \frac{1}{s}} \right)}} \left( {\lambda  - t} \right)} \right]} \right\}^{{\left( { - \frac{1}{s}} \right)}} } & {{\text{Richards}}}  \\   \end{array} } \right. $$

According to the mathematical equations, we used parameters *λ* (lag time), *R* (maximum specific growth rate) and *A* (asymptotic growth) to describe three continuous phases of bacterial growth curves, respectively. Moreover, these three key parameters were used as phenotypic data in GWAS analyses. In the Richards equation *s* is a shape parameter that describes the curvature of a growth curve (Wei et al. 2018).

First, we used all three growth equations to fit *E. coli* and *S. aureus* monoculture growth trait data at each location using a nonlinear least squares approach. Then we calculated each equation's AIC value. This procedure allows the choice of an optimal growth equation that best fits bacterial growth at a given location, dependent on statistical reasoning. The three parameters mentioned above were estimated from the optimal equation and used as phenotypic traits for subsequent functional mapping.

### GWAS model

We analyzed the associations between SNPs and microbial abundances measured for bacterial populations reared in monocultures and co-cultures. We calculated the log_10_ (*P*-values) of each association, from which Manhattan plots were derived.

In co-cultures, we used binary correlation coefficients to treat the three parameters as three different periods of phenotypic data combined with genotype data for comparative analyses. We constructed two-dimensional Manhattan plots, thereby obtaining significant SNPs of interaction between the two strains under the same culture conditions.

## Results

### Data fitting by the growth equations

**Fig. 1:**
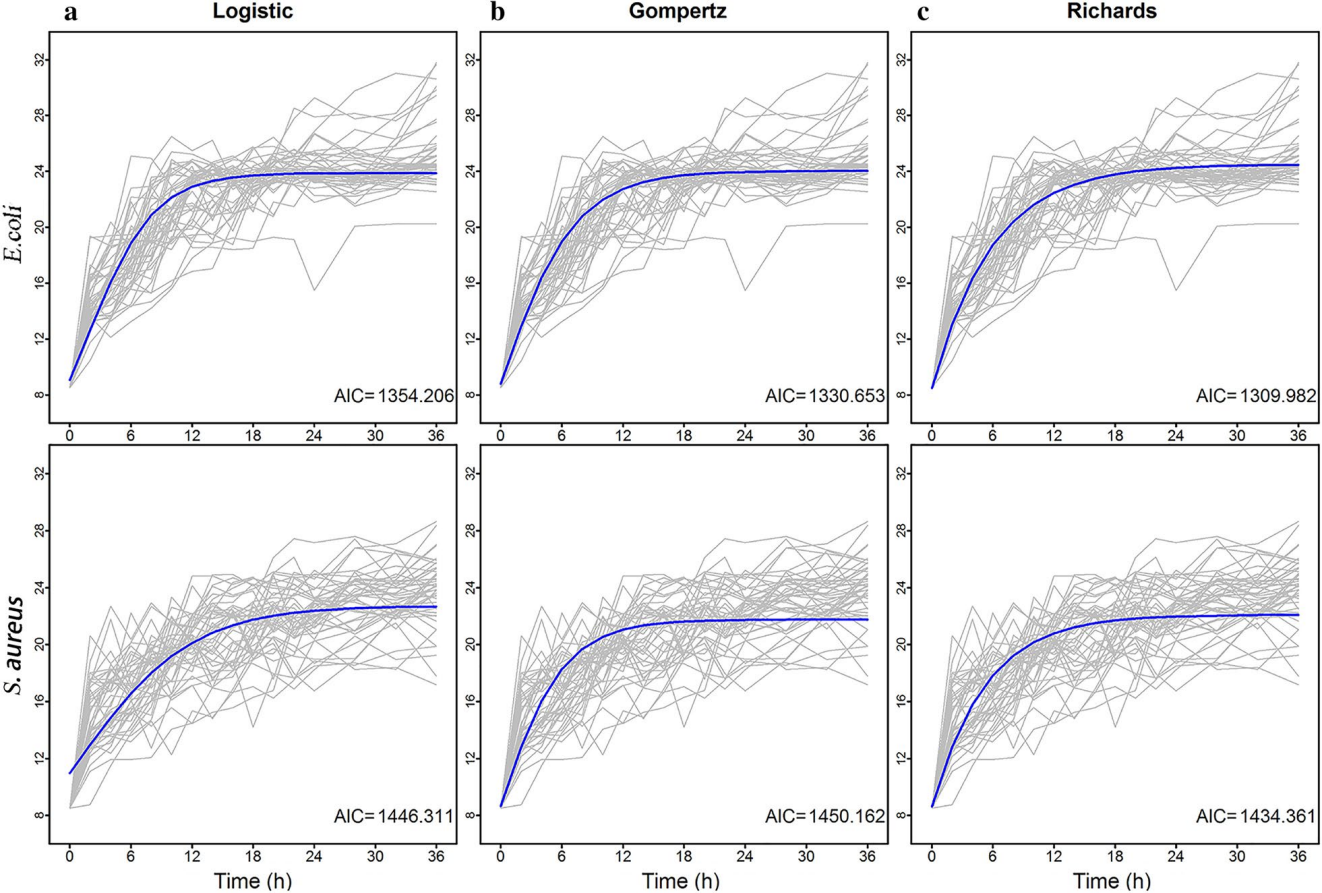
Fitting growth curves of *E. coli* and *S. aureus* phenotypic data under three equations. **a** Logistic; **b** Gompertz; **c** Richards

Three commonly used regression equations of growth, namely, Logistic, Gompertz and Richards regressions, were used to fit the growth curves of 45 *E. coli* and 45 *S. aureus* strains (Fig. 1 and Additional file 3: Figure S1). Microbial abundance of individual strains was observed over 36 h. An optimal equation was chosen based on Akaike’s information criterion (AIC). Richards regression had a better goodness-of-fit to the mean growth trajectories and thus was used in analyses of phenotypic data and to fit co-culture bacterial growth curves.

### Genetic analyses of microbial growth in monocultures

**Fig. 2:**
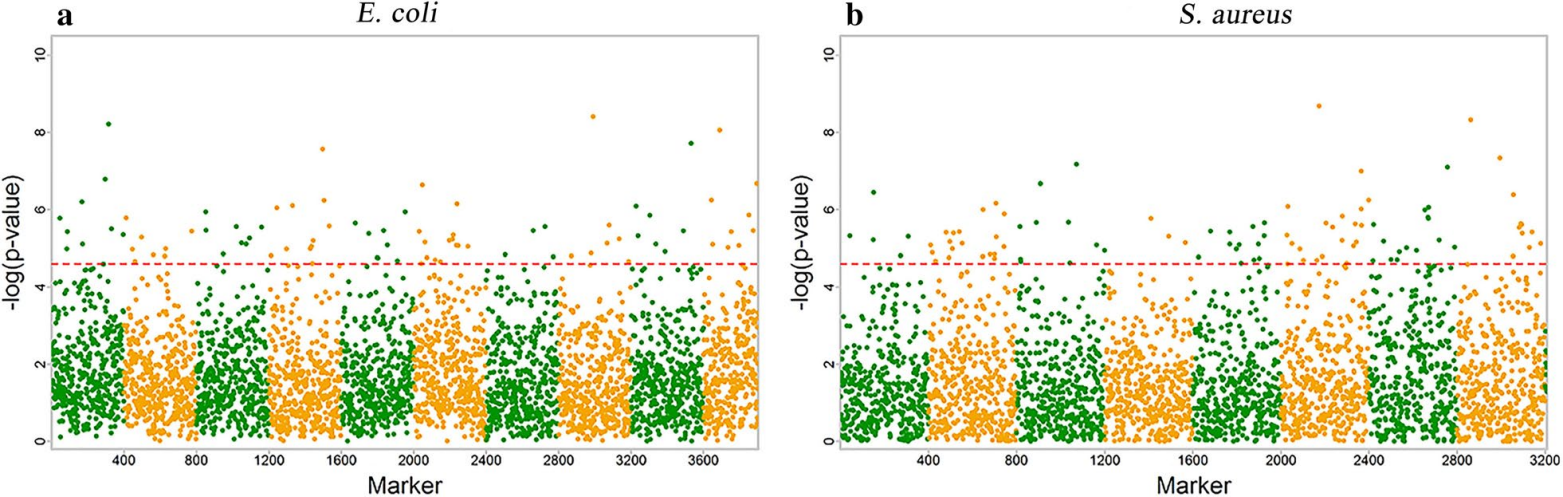
Manhattan plots of GWAS results for *E. coli* and *S. aureus* in monoculture. **a** *E. coli*; **b** *S. aureus*

Manhattan plots were obtained by employing GWAS combined with functional mapping of 45 *E. coli* and 45 *S. aureus* strains to identify polymorphisms associated with different growth phenotypes. Figure 2 shows Manhattan plots for significant SNPs identified in monocultures of each strain. In all, 85 and 97 significant SNPs were beyond the genome-wide critical thresholds determined for *E. coli* and *S. aureus*, respectively, and were annotated. Of those, 71 SNPs in *E. coli* mapped to 67 genes, were associated with growth; 63 SNPs in *S. aureus*, were mapped to 45 genes (Additional file 4: Table S3). Interestingly, many of the significant SNPs detected in GWAS were distributed in the genomic regions involved in metabolism and regulation.

**Fig. 3:**
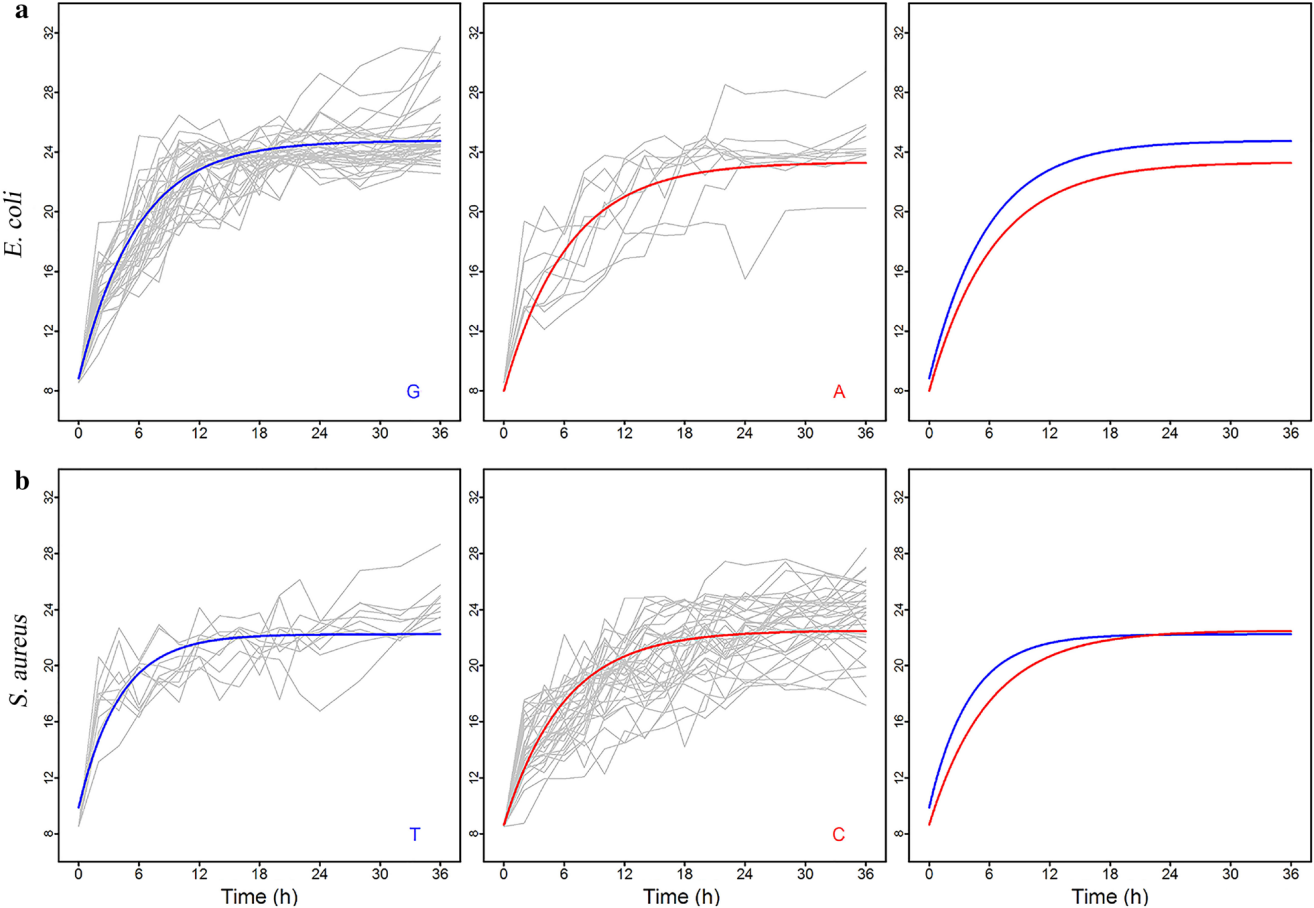
Fitting growth curves of the most significant SNPs of *E. coli* (E3393816) and *S. aureus* (S994124). **a** *E. coli*; **b** *S. aureus*

To demonstrate the biological relevance of the model, we cultured all strains individually in isolated flasks. The microbial abundance of each strain was fitted separately for two alternative genotypes at E3393816 for *E. coli* and at S994124 for *S. aureus* (Fig. 3). We found different phenotypic data for each genotype. In *E. coli*, G genotype strains grew relatively faster than A genotype strains; in *S. aureus*, T genotype strains grew faster than C genotype strains in the exponential phase but grew uniformly in the stationary phase. Therefore, differences in genotypes affect the growth of strains, resulting in phenotypic diversity.

### Interaction analyses based on growth parameters in co-culture

**Fig. 4:**
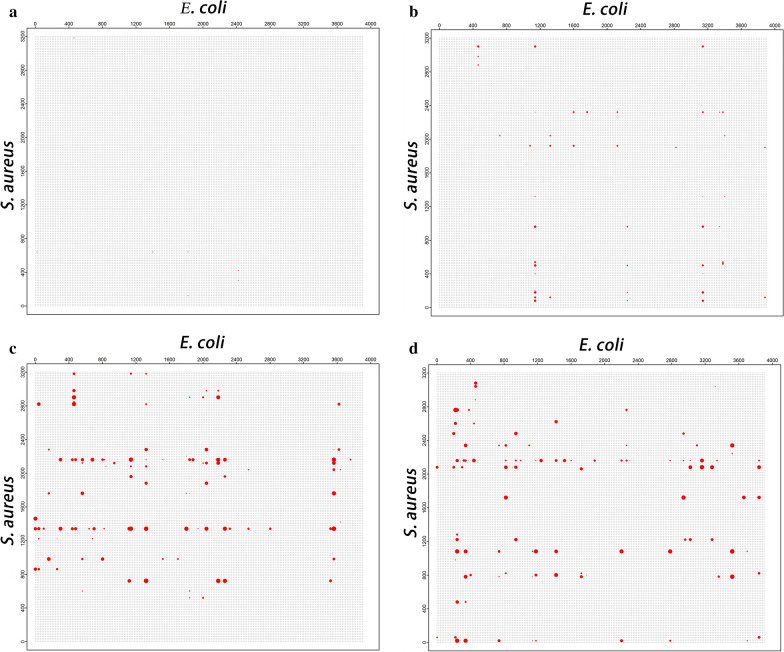
Two-dimensional Manhattan plots based on growth parameters of *E. coli* and *S. aureus* in co-culture. **a** *λ* parameter; **b** *R* parameter; **c** *A* parameter; **d** *s* parameter

In co-culture we used the Richards equation to fit bacterial growth and the curve fitting for growth data of each strain under co-culture were shown in Additional file 3: Figure S1. The four biologically meaningful growth parameters, λ, *R*, *A* and *s*, estimated from the Richards equation, were each used as a ‘phenotypic trait’ for subsequent GWAS analysis. We performed GWAS based on growth parameters to identify significant SNPs between the genotype and phenotype data of *E. coli* and *S. aureus*, and combined with the R package to generate the two-dimensional Manhattan plots (Fig. 4). The x-axis represents the relative SNP position of *E. coli*, the y-axis represents the relative SNP position of *S. aureus*, and the red dots in the figure represent the significant SNPs affecting the bacterial interaction. According to the corresponding positions of SNPs in the two-dimensional plots, their positions in the genome can be identified and then the significant SNPs in the interaction can be annotated. The R^2^ value of fitting growth curves of *E. coli* and *S. aureus* in co-culture were shown in Additional file 5: Table S4.

### Analyses of significant genes in *E. coli*

In *E. coli*, there were 5 significant SNPs under the lambda (*λ*) parameter and 12 under the *R* parameter. Thirty-four significant SNPs were under the *A* parameter and shape (*s*) parameters, respectively (Additional file 6: Table S5). Among the significant SNPs in *E. coli*, eight genes were associated with significant functions in bacterial growth: *aceE* encodes a pyruvate dehydrogenase E_1_ component that plays an important role in the preparation stage of the citric acid cycle; *yjjW* encodes a homolog of pyruvate formate lyase activating enzyme PflA; *dnaK* functions in the heat shock response; *ftsK* and *ftsA* are relevant to cell division*; tatD* encodes 3′ to 5′ ssDNA/RNA exonuclease*;* the protein which *rclR* encodes is a transcriptional activator*,* an*d fepA* effects ferric enterobactin outer membrane transporter (Table 1).

**Table 1:** Significantly important functional genes in co-culture

Gene ID	Gene	Position	Mutation	Affected Codon	Function
*E. coli*					
B0114	*aceE*	124522	T↔C	GCT↔GCC	Pyruvate dehydrogenase E_1_ component
B4379	*yjjW*	4614704	C↔T	TTG↔TTA	Putative glycyl-radical enzyme activating enzyme YjjW
B0014	*dnaK*	12384	T↔C	GGT↔GGC	Chaperone protein DnaK
B0094	*ftsA*	104986	C↔T	ACC↔ACT	Cell division protein FtsA
B0890	*ftsK*	935503	G↔A	CCG↔GCA	Cell division DNA translocase FtsK
B4483	*tatD*	4024120	T↔G	CTT↔TTG	3′→5′ ssDNA/RNA Exonuclease TatD
B0584	*fepA*	611271	T↔C	GGA↔GGG;	Ferric enterobactin outer membrane transporter
B0305	*rclR*	320655	A↔T	GCA↔GCT	DNA-binding transcriptional activator RclR
*S. aureus*					
SAOUHSC_00299	*scdA*	313,151	C↔G	AGC↔ACC	Cell wall biosynthesis protein ScdA
SAOUHSC_01368	*trpD*	1,313,228	T↔A	TCC↔ACC	Anthranilate phosphoribosyltransferase
SAOUHSC_00545	*sdrD*	554,869	C↔T	GAC↔GAT	Fibrinogen-binding protein SdrD
SAOUHSC_01219	-	1,169,344	G↔C	GAT↔CAT	Cell wall hydrolase

### Analyses of significant genes in *S. aureus*

Meanwhile, in *S. aureus*, there were three significant SNPs under the lambda (*λ*) parameter, 13 under the *R* parameter, and seventeen under the shape (*s*) parameter. *A* parameter SNPs were the most diverse, with 19 significant SNPs (Additional file 6: Table S5). In *S. aureus*, we found many significant genes, such as *scdA* encoding a cell wall biosynthesis protein, and *sdrD* encoding a fibrinogen-binding protein. A hypothetical gene (SAOUHSC_01219) was predicted for the encoded cell wall hydrolase (Table 1). Many significant genes were found to have an important role in the regulation of metabolism and bacterial reproduction and regulation, which may be correlated with evolution or growth performance in co-culture.

## Discussion

Bacterial interaction plays a vital role in the ecosystems (Rivett and Bell 2018). Previous studies illustrated that the changes of phenotypes in bacterial interaction were related to complex systems (such as quorum sensing), and cannot be predicted from analyzing the individual bacteria strain (Madsen et al. 2018). Many researches had used GWAS to unravel the genetic machineries of interspecies interactions in microbes (Berthenet et al. 2018; Collins and Didelot 2018; He et al. 2017; Rong et al. 2019). Wei et al. (2018) found that identification of major pleiotropic QTLs for leaf growth trajectories can be performed based on a dynamic mapping model, which has not been applied to bacteria yet. Therefore, in this study we extended the application of this model, which is based on growth parameters of GWAS, to the study of interactions between *E. coli* and *S. aureus*.

In this research, we found some significant genes such as *yjjW, thrC, kefC, ftsA, wzc* and *yeiI*, which were reported in previous studies (He et al. 2017; Jiang et al. 2018). Besides, more significant genes were identified in this study including *dnaK, tatD, rclR, ftsA, ftsK, aceE, fepA* in *E. coli*, and *sdrD*, *scdA* in *S. aureus*. Gene *dnaK* encodes ATP-dependent enzyme DnaK and plays an important role in the heat shock response (Collet et al. 2018). The stress response is not the only function of DnaK, which also has a significant role in maintaining normal growth in *E.coli* (Ghazaei 2017). Gene *tatD* encodes DNA-repairing exonuclease that not only digest chromosomal DNA during apoptosis but also process damaged DNA during DNA repair. Chen et al. (2014) demonstrated that TatD-knockout strains were less resistant to the DNA damaging and were sensitive to H_2_O_2_. Gene *rclR* encodes RclR as a transcriptional activator, contributing to the ability of *E. coli* to survive HOCl stress (Parker et al. 2013). These three genes can regulate bacterial activities when they are in a negative environment. In addition, there are many genes that play important roles in cell growth and metabolism, such as *ftsA* and *ftsK* encodes FtsA and FtsK, respectively. FtsK is present as a hexamers and plays a key role in coordinating cell division in the late stages of chromosome segregation and FtsA is an ATPase (Bisicchia et al. 2013; Conti et al. 2018; Galli et al. 2017), both of which assist FtsZ in cell division. Gene *aceE* encodes the E_1_ component of pyruvate dehydrogenase complex. The E_1_ component plays a role in the preparation phase of pyruvic acid before which enters the citric acid cycle and catalyzes the oxidative decarboxylation of pyruvate (Byung Jo et al. 2008; Nemeria et al. 2010). FepA plays a role in the transporter activity of the ferric enterobactin outer membrane, which is located in the lipid bilayer of the outer membrane of *E. coli* and belongs to the outer membrane protein of the cell wall. Its main function is to adsorb high-valent iron on the cell surface when *E. coli* is iron-deficient (Newton et al. 2010; Turlin et al. 2013). In *S. aureus*, *sdrD* encodes fibrinogen-binding protein serine aspartate repeat containing protein D (SdrD) (Askarian et al. 2016). The *scdA* gene expresses a cell wall biosynthesis protein that affects cell division and morphogenesis (Brunskil et al. 1997).

However, significant genes reported from this interaction analyses based on growth parameters of GWAS still require functional validation in our following research. With the application of CRISPR/Cas9 technology to bacteria in recent years (Banno et al. 2018; Chen et al. 2017, 2018; Zerbini et al. 2017), this study will be improved greatly. By constructing recombinant plasmids for knockout and mutation of target genes, it will assist in comparing phenotypic differences between wild-type and mutants to validate the effects of genes on growth. Next, we will apply CRISPR/Cas9 to verify those function genes explored in the interaction analyses.

## Supplementary Information

**Additional file 1: Table S1.** Strain IDs.

**Additional file 2: Table S2.** Table S2 Growth performance of *E. coli* and *S. aureus*.

**Additional file 3: Figure S1.** The curve fitting for growth data of E. coli (a) and S. aureus (b) in co-culture.

**Additional file 4: Table S3.** Gene annotation of *E. coli* and *S. aureus* in monoculture.

**Additional file 5: Table S4.** Fitting R^2^ of growth curves in *E. coli* and *S. aureus* in co-culture.

**Additional file 6: Table S5.** Gene annotation of *E. coli* and *S. aureus* in co-culture.

## Data Availability

Data sequences: the raw sequence data of *E. coli* generated in this study were deposited in the NCBI Gene Expression Omnibus under Accession No. SRP074089 and the data of *S. aureus* under Accession No. SRP074912.
