# Supplementary material for: Interaction analyses based on growth parameters of GWAS between Escherichia coli and Staphylococcus aureus
Source: AMB Express. 2021 Mar 1;11:34. doi: 10.1186/s13568-021-01192-x (PMC7921238; doi:10.1186/s13568-021-01192-x)
Supplement: Supplementary file 5 — Additional file 5: Table S4. Fitting R2 of growth curves in E. coli and S. aureus in co-culture. [file 13568_2021_1192_MOESM5_ESM.docx]

Table S4 Fitting R^2^ of growth curves in *E. coli* and *S. aureus* in co-culture

| **Number** | ***E. coli*** | ***S. aureus*** | **Number** | ***E. coli*** | ***S. aureus*** |
| --- | --- | --- | --- | --- | --- |
| 1 | 0.9542 | 0.9664 | 26 | 0.9766 | 0.9215 |
| 2 | 0.9496 | 0.933 | 27 | 0.98 | 0.8689 |
| 3 | 0.963 | 0.9102 | 28 | 0.9815 | 0.8123 |
| 4 | 0.9494 | 0.9347 | 29 | 0.9665 | 0.9105 |
| 5 | 0.9812 | 0.8765 | 30 | 0.9947 | 0.9239 |
| 6 | 0.9855 | 0.8892 | 31 | 0.9854 | 0.9652 |
| 7 | 0.9943 | 0.9862 | 32 | 0.9908 | 0.9862 |
| 8 | 0.986 | 0.8603 | 33 | 0.9213 | 0.9861 |
| 9 | 0.9839 | 0.9529 | 34 | 0.9786 | 0.9168 |
| 10 | 0.9774 | 0.8796 | 35 | 0.9693 | 0.9347 |
| 11 | 0.9667 | 0.9548 | 36 | 0.9901 | 0.9208 |
| 12 | 0.9653 | 0.9213 | 37 | 0.9873 | 0.9335 |
| 13 | 0.9751 | 0.9088 | 38 | 0.9735 | 0.8719 |
| 14 | 0.9845 | 0.9144 | 39 | 0.9801 | 0.9056 |
| 15 | 0.9713 | 0.9412 | 40 | 0.9911 | 0.9353 |
| 16 | 0.9344 | 0.9894 | 41 | 0.9794 | 0.964 |
| 17 | 0.9023 | 0.9842 | 42 | 0.9837 | 0.9171 |
| 18 | 0.9424 | 0.983 | 43 | 0.979 | 0.9732 |
| 19 | 09131 | 0.9858 | 44 | 0.9802 | 0.9647 |
| 20 | 0.9253 | 0.9898 | 45 | 0.9901 | 0.9447 |
| 21 | 0.9406 | 0.9609 |  |  |  |
| 22 | 0.9478 | 0.9636 |  |  |  |
| 23 | 0.9499 | 0.8505 |  |  |  |
| 24 | 0.934 | 0.8746 |  |  |  |
| 25 | 0.8941 | 0.8892 |  |  |  |
